# Comprehensive chemical profiling of *Bassia indica* Wight. aerial parts extract using UPLC-ESI–MS/MS, and its antiparasitic activity in *Trichinella spiralis* infected mice: in silico supported in vivo study

**DOI:** 10.1186/s12906-023-03988-9

**Published:** 2023-05-18

**Authors:** Magdy M. D. Mohammed, Elham A. Heikal, Fatma M. Ellessy, Tarek Aboushousha, Mosad A. Ghareeb

**Affiliations:** 1 Pharmacognosy Department, Pharmaceutical and Drug Industries Research Institute, National Research Centre, Dokki-12622 Cairo, Egypt; 2 Department of Medical Parasitology, Faculty of Medicine, Al-Azhar University, Cairo, Egypt; 3 Department of Pathology, Theodor Bilharz Research Institute, Kornaish El-Nile, Warrak El-Hadar, Imbaba, (P.O. 30), Giza, 12411 Egypt; 4 Medicinal Chemistry Department, Theodor Bilharz Research Institute, Kornaish El-Nile, Warrak El-Hadar, Imbaba, (P.O. 30), Giza, 12411 Egypt

**Keywords:** *Bassia indica*, Chenopodiaceae, *Trichinella spiralis*, Saponins, Phenolics, Molecular Docking

## Abstract

**Background:**

*Trichinellosis* is a public health threat infected both animals and humans as a result of eating undercooked meat. It caused by *Trichinella spiralis* that has widespread drug resistance and even developed many sophisticated strategies for their survival, this increases the demand in searching for new anthelmintic drugs from natural source.

**Methods:**

Our objectives were to test the in vitro and in vivo anthelmintic activity of *Bassia indica* BuOH frac., and to characterize its chemical composition using UPLC-ESI–MS/MS. Besides an in silico molecular docking study with the prediction of the PreADMET properties.

**Results:**

In vitro investigation of *B. indica* BuOH frac., showed severe destruction of the adult worm and larvae, marked cuticle swelling, areas with vesicles, blebs and loss of annulations. This was assured via in vivo study, which revealed a significant reduction (*P* < 0.05) in the mean adult worm count with efficacy of 47.8% along with a significant decrease (*P* < 0.001) in the mean larval count per gram muscle with efficacy 80.7%. Histopathological examinations of the small intestine and muscular sections showed marked improvement. In addition, immunohistochemical findings demonstrated that *B. indica* BuOH frac. depressed the proinflammatory cytokines expressions of TNF-*α*, which was obviously upregulated by *T. spiralis.* Precise chemical investigation of the BuOH frac. using UPLC-ESI–MS/MS resulted in the identification of **13** oleanolic type triterpenoid saponins; oleanolic acid 3-*O*-6´-*O*-methyl-*β*-D-glucurono-pyranoside (**1**), chikusetsusaponin-IVa (**2**) and its methyl ester (**3**), chikusetsusaponin IV (**4**) and its methyl ester (**5**), momordin-Ic (**6**) and its methyl ester (**7**), betavulgaroside-I (**8**), -II (**9**) -IV (**10**), -X (**11**), licorice-saponin-C_2_ (**12**) and -J_2_ (**13**). In addition, **6** more phenolics were identified as syringaresinol (**14**), 3,4-di-*O*-caffeoylquinic acid (**15**), 3-*O*-caffeoyl-4-*O*-dihydrocaffeoylquinic acid (**16**), 3,4-di-*O*-caffeoylquinic acid butyl ester (**17**), 3,5-di-*O*-galloyl-4-*O*-digalloylquinic acid (**18**) and quercetin 3-*O*-(6´´-feruloyl)-sophoroside (**19**). The auspicious anthelmintic activity was further ascertained using in silico molecular docking approach that targeted certain protein receptors (*β*-tubulin monomer, tumor necrosis factor alpha (TNF-*α*), cysteine protease (*Ts*-CF1), calreticulin protein (*Ts*-CRT)), all the docked compounds (**1–19**) fit into the binding site of the active pocket with binding affinities noteworthy than albendazole. In addition, ADMET properties, drug score and drug likeness were predicted for all compounds.

**Supplementary Information:**

The online version contains supplementary material available at 10.1186/s12906-023-03988-9.

## Introduction

*Trichinellosis*, a widespread zoonoses, caused by *T. spiralis* a food-borne parasitic nematode, which is gained via the ingestion of undercooked meat ´mainly pork` and/or its products infected with *T. spiralis* encysted larvae [1]. *Trichinellosis* considered to be a public health threat, that infected around 11 million globally based on its wide range of hosts that include animals (150 species) and even humans [2, 3]. World Health Organization (WHO) and the Agriculture Organization (FAO) of the United Nations (UN) considered *T. spiralis* is the first ranked among 24 parasites in international trade [4]. Cysteine proteases derived from parasite play a ticklish role in pathogenesis involved in tissue penetration, molting, nutrition metabolism, immune evasion and/or modulation [5]. Recently, a cysteine protease of *T. spiralis* (*Ts*-CF1) was expressed in its life stages and localized in the cuticle and stichosome, which favored its potential use as a drug target and/or vaccine candidate against *T. spiralis* infection [6]. The life cycle of *T. spiralis* composed of three stages (adult, newborn larvae ´NBL`, and infective muscle larvae ´ML`), during which, and to escape host innate and adaptive immune attack, it establishes a range of immune evasion strategies. One of its sophisticated mechanisms is the secretion of functional proteins *i.e.,* calreticulin (*Ts*-CRT), that overlaps, binds, and interferes with the host classical complement immune system, and hence facilitate their survival in the hostile host [7].

Human *trichinellosis* generates a broad spectrum of symptoms including diarrhoea, fever, myalgia and periorbital oedema, the approved treatment with benzimidazole-2-carbamate derivatives (**BzC**) such as albendazole (**ABZ**), mebendazole (**MBZ**), flubendazole (**FBZ**), etc. [8], which are selectively bind to the *β*-tubulin monomer of the parasite and inhibit its microtubule polymerization, but with little effect due to binding tubulin of the mammalian host [9, 10]. However, they are still not fully effective against encysted or newborn larvae, because of their low bioavailability, drug resistance and they are not recommended for children and are contraindicated during pregnancy [3]. Nevertheless, continuous development of helminths resistance against synthetic anthelmintic drugs encourage the evaluation of medicinal plants as an alternative source for anthelmintics. Moreover, plant-based medicines are generally more attainable and affordable [11].

Currently, the use of medicinal plants extracts against parasitic and/or non-parasitic infection have been handled by many studies, based on their ethnopharmacological use as anti-bacterial infection, rheumatic, gastro-intestinal parasite infections, diarrhoea, dysentery, respiratory diseases and cough [12]. *B. indica* Wight. (Syn. *Kochia indica*) an Egyptian medicinal plant belongs to the family Chenopodiaceae, it is native to India and widely distributed in the Western Mediterranean to Eastern Asia [13]. *Bassia* species are rich in phytoconstituents including: sterols, alkaloids, flavonoids and saponins [14–16], and it have been reported to treat variety of ailments including analgesic, anti-inflammatory, antiallergic, antipyretic, anticancer and antiviral [16–18], and as anti-bacterial, antifungal, antiparasitic [19]. Recently, Aboul-Enein and co-workers examined the in vitro anticancer activity of *K. indica* Wight (a syn. of *B. indica* Wight) water and ethanol extract (EtOH ext.) against the cell growth of EACC at the 100* μg*/mL concentration, with complete inhibition at 2.88% and 1.60% respectively [20], in addition, it recorded antioxidant activity against DPPH with of 50.40% and 72.40% respectively. Moreover, Bibi and colleagues inspected the in vitro antibacterial activity of *K. indica* Wight (a syn. of *B. indica* Wight) EtOH ext. at different concentrations against *S. typhi, E. coli, S. flexneri, P. aeroginosa* and* S. aureus* strains and resulted with 13.40%—20.37% (at 15 mg/mL), 13.11%—19.10% (at 10 mg/mL) and 11.20%—17. 44%, (at 5 mg/mL) concentration over negative control [21]. Furthermore, Javed and companions reported a significant inhibitory activity of *K. indica* Wight (a syn. of *B. indica* Wight) n-BuOH frac. against *Macrophomina phaseolina* with 63–92% reduction in its biomass [22]. In addition, the in vitro neuroprotective effects of the crude extracts and amide alkaloids isolated from *B. indica* Wight were reported with potential activity which was confirmed via in silico study [16].

Therefore, with this hypothesis, our present study intended to characterize the saponins and phenolics content of *B. indica* aerial parts using UPLC-ESI–MS/MS, and to investigate its in vitro and in vivo anthelminthic activity against *T. spiralis*, which is supported by a detailed in silico modelled study.

## Materials and methods

All methods were carried out in accordance with relevant guidelines.

### Plant material

The *B. indica* Wight. aerial parts were collected after permission and in compliance with relevant international guidelines and legislation from Port Tawfik, Port Said Governorate, Egypt during June 2020. The plant materials were authenticated by Mrs. Therese Labib, consultant of taxonomy at the ministry of agriculture, and the former director of El-Orman Botanical Garden, Giza, Egypt. A voucher specimen no (1–12-2) of the plant was deposited at the Herbarium of El-Orman Botanical Garden.

### Extraction and fractionation

*B. indica* aerial parts (1.5 kg) were air dried in shade, powdered into fine particles and then extracted by maceration using 70% methanol (MeOH) at r.t. till exhaustion, the resulted crude ext., was successively fractionated using liquid/liquid partitioning, including defatting with pet. Ether (60–80 °C), chloroform/ethyl acetate (CHCl_3_/EtOAc), *n*-BuOH, and H_2_O. All fractions were investigated for the presence of triterpenoidal saponins and/or phenolics using 2D-PC (Whatman® 3MM) and TLC (Kieselgel 60 F_254_, aluminum backed, Merck, Darmstadt, Germany). The obtained results favored *n*-BuOH fraction (19.83 g) over the others, which developed pink to purple colored spots upon spraying with vanillin-H_2_SO_4_ characteristic for triterpenoids. In addition, spraying with AlCl_3_ and Natural Product Reagent (NPR), changes the colored spots from quenching fluorescence to yellow, orange or blue, typical for flavonoids, phenolics and/or their acids, at 366 nm [23]. Hence, the *n*-BuOH fraction was subjected to UPLC-ESI–MS/MS advanced analysis for the identification of its major constituents, as well as to study its biological potential.

### UPLC-ESI–MS/MS conditions

UPLC-ESI–MS/MS analyses (± ve modes) were also performed on the UPLC system (Waters Acquity BEH, MA-01757, USA) coupled with an electrospray mass spectrometer. Mass spectrometry was carried out on a Waters triple-quadrupole mass spectrometer (Waters Acquity, XEVO, TQD, MA, USA). The LC effluent entered the mass spectrometer through an electrospray capillary set at + 3.00 kV, with a source temperature of 150ºC. Nitrogen was used as cone and desolvation gas at flow rates of 50 & 900L/h, respectively, with desolvation temp. 440ºC. Chromatographic separation was conducted by injecting 0.5*μ*L of the extract (post filtering using 0.2* μm* filter membrane disc and degassed by sonication before injection.) on a C_18_ (50 × 2.1 mm, 1.7* µm* particle size) column (Waters). Gradient elution was achieved using the solvents (A) 0.1% (v/v) formic acid in water, and (B) 0.1% formic acid in acetonitrile at a flow rate of 0.2 mL/min, the linear gradient program gave the optimal sensitivity. Mass spectra were detected in the ESI between *m/z* 100–1000, peaks and spectra were processed using the MassLynx 4.1 software and tentatively identified by comparing its retention time (R_*t*_) and mass spectrum fragmentations with reported data from the literature and the Wiley natural product library for the structural identifications of phenolic compounds as well as triterpenes.

### Assessment of anti-anthelmintic activity

#### Animals, parasites and materials

Swiss albino male mice aged 5–6 weeks and weighing 20–25 g at the beginning of the experiment were obtained from Schistosome Biological Supply Centre (SBSC), Theodor Bilharz Research Institute (TBRI) (Giza, Egypt). The mice were kept on a standard commercial pelleted diet with free accessible water and ensuring good sanitary conditions throughout the time of the study. The strain of *T. spiralis* was obtained from the Parasitology department, TBRI. Mice were orally infected with 200 *T. spiralis* larvae [24]. Albendazole was purchased as Bendax tablets (200 mg) from Sigma Pharmaceutical Industries, Egypt.

#### Isolation of *T. spiralis* adult worms and muscle larvae

*T. spiralis* adults and muscle larvae were obtained from the infected mice as reported [25]. Swiss albino mice infected with *T. spiralis* for 30 days were sacrificed, muscles were separated and minced, and muscle larvae were digested by immersion in the acid pepsin solution [26]. The mixture was incubated at 37 °C for 2 h subjected to continuous stirring using an electric stirrer, followed by filtration of the digest [27]. The collected larvae were washed two to three times with tap water and suspended in a conical flask for half an hour to allow sedimentation. *T. spiralis* adult worms were isolated from the small intestines of infected untreated mice six days post-infection (p.i.), briefly, the intestine was washed, opened longitudinally along its entire length, cut into small pieces 2 cm each and placed in normal saline at 37 °C for three to four hours to allow the worms to migrate out of the tissue [28].

#### In vitro experimental study

*T. spiralis* adult worms (30 parasites per well) were cultured in a 24-well tissue culture plate prepared with an incubation medium consisting of RPMI-1640 Medium (containing 20% fetal bovine serum, 200U/mL penicillin and 200* μg*/mL streptomycin). Six groups were established in this study as follow: Group–I: adult worms cultured in an incubation medium only, Group–II: adult worms cultured in an incubation medium containing *B. indica* BuOH frac., dissolved in dimethyl sulfoxide (DMSO) at a concentration of 100* μg*/mL, Group–III: adult worms cultured in an incubation medium containing albendazole that was dissolved in DMSO at a concentration of 100* μg*/mL [29], Group–IV: larvae of *T. spiralis* cultured in an incubation medium only, Group–V: larvae of *T. spiralis* cultured in an incubation medium containing *B. indica* BuOH frac., Group–VI: larvae of *T. spiralis* were cultured in an incubation medium containing albendazole dissolved in DMSO at a concentration of 100* μg*/mL, three wells were used for each group, and the plate was placed in the incubator at 37 °C and 5% carbon dioxide for 24 h.

#### Scanning electron microscopy (SEM)

Adult worms and larvae were processed after 24 h [30], briefly, worms from each group were directly pipetted and immediately fixed in a fresh fixation solution of 2.5% glutaraldehyde solution buffered with 0.1 M sodium cacodylate at pH 7.2 and left overnight at 4 °C. The fixed specimens were then washed in 0.1 M sodium cacodylate buffer at pH 7.2 for 5 min, post-fixed in 2% osmium tetroxide for 1 h and washed in distilled water. The specimens were dehydrated in ascending grades of ethyl alcohol and then mounted on carbon-coated adhesive material and examined using a scanning electron microscope (Jeol GSM- IT200, Japan), Electronic Microscope Unit, Faculty of Science, Alexandria University.

### In vivo experimental study

#### Study design and ethics

Swiss albino male mice (*n* = 108) aged 5–6 weeks and weighing 20–25 g at the beginning of the experiment were obtained from the Schistosome Biological Supply Centre (SBSC), Theodor Bilharz Research Institute (TBRI), Giza, Egypt. The mice were kept on a standard commercial pelleted diet with free accessible water and ensuring good sanitary conditions throughout the time of the study. Handling, anesthetic and sacrifice procedures followed ethical guidelines approved by the Ethical Committee of the Federal Legislation, the National Institutes of Health Guidelines in the United States and approved by the Research Ethics Committee of the Faculty of Medicine for Girls, Al Azhar University, Egypt, for the conduct of animal experiments (Approval No. 2022061392).

In the in vivo study, mice were divided into six groups of 18 mice each: Group-I: non-infected non-treated (control negative). Group-II: non-infected treated, receiving *B. indica* BuOH frac. Group-III: infected non-treated (control positive). Group-IV: receiving *B. indica* BuOH frac., for seven days before infection (as prophylaxis). Group-V: infected and treated with *B. indica* BuOH frac. (given orally at a dose of 100 mg/kg) and dissolved in distilled water [31], Group-VI: infected and treated with albendazole (given orally at a 50 mg/kg dose and suspended in 10% Tween 80 and 90% deionized water) just before oral administration [32]. Groups -V & -VI were further divided into three subgroups (a, b and c); each of which was comprised of six animals each, in order to assess the effect of the drugs given during the intestinal phase only–(a) (3–5 days p.i.), muscular phase only–(b) (30–32 days p.i.), and intestinal then muscular phases–(c) (3–5 days p.i. and 30–32 days p.i.) separately.

#### Detection of* T. spiralis* adult worms and muscle larvae burden

On the scheduled termination day, all mice were fasted overnight and sacrificed by anesthesia intraperitoneally using urethane (Sigma-Aldrich, St Louis, MO; 1.3–1.5 g/kg in a ~ 1.5 g/5 mL 0.9% normal saline solution). In subgroups–a (intestinal phase), scarification of mice was done on day 6 p.i., and small intestine was processed as previously described, *T. spiralis* adults were obtained, and the worm reduction rate was calculated. Moreover, in subgroups–b (muscular phase), the mice were sacrificed on day 35 p.i., and muscle larvae were obtained by pepsin digestion method. The larvae were counted microscopically using the McMaster counting chamber, Faust-Germany. Parasite burdens were expressed as the number of larvae per gram of carcass digested (ML/g) [33].

#### Histopathological study

Parts of the intestine and skeletal muscles from the studied groups were fixed in 10% formalin for 24 h, washed in water for 12 h, and dehydrated in ascending grades of alcohols and cleared in xylene. Impregnation was done in pure soft paraffin for 2 h at 55 °C. Then, hard paraffin sections of 5* μm* thickness were cut by microtome. Sections were stained with haematoxylin and eosin stain [34]. Sections from the intestine were histopathologically examined for presence or absence of *Trichinella* larva, any villous abnormality and intensity of inflammatory cellular reaction. Sections from skeletal muscles were examined for the presence of *Trichinella* cysts, counted, and comments up on the integrity of the capsule, the content and the inflammatory cellular reaction were reported in each group.

#### Immunohistochemical (IHC) technique

Paraffin sections from skeletal muscle specimens underwent de-paraffinization and rehydration. Antigen retrieval was performed by microwaving the sections in citrate buffer, pH 6.0. Endogenous peroxidase was blocked with methanol containing 3% hydrogen peroxide. Sections were incubated overnight at 4 °C in humid chamber with the primary antibodies: Anti-CD34 Antibody (Clone: QBEnd-10, Mouse anti-Human, Supplier: DAKO, Catalog Number: M7165), in an optimal dilution of 1:50–1:100, followed by application of secondary antibody (Biotin-streptavidin link, DAKO). Then, the antigen was localized by the addition of 3,3´-diaminobenzidine tetrahydrochloride (DAB) substrate chromogen solution (Universal Detection Kit, DAKO Envision, Denmark). Finally, slides were counterstained with hematoxylin, dehydrated in alcohol and mounted. For each setting, positive and negative control slides were included. As a negative control, skeletal muscle tissue was processed in the above-mentioned sequences, but the primary antibodies were not added and instead add non-immune immunoglobulin G (IgG; DAKO, Glostrup, Copenhagen, Denmark).

#### Interpretation of immunostaining and scoring statistical analysis

The data were analyzed using Microsoft Excel 2016 and statistical package for social science IBM SPSS Statistics for Windows, version 26 (IBM Corp., Armonk, New York, USA). Continuous normally distributed variables were represented as mean ± standard deviation, a *P*-value < 0.05 will be considered statistically significant. Student’s t-test was performed to compare the means of normally distributed variables between groups were performed.

#### In silico molecular docking study

**Fig. 1:**
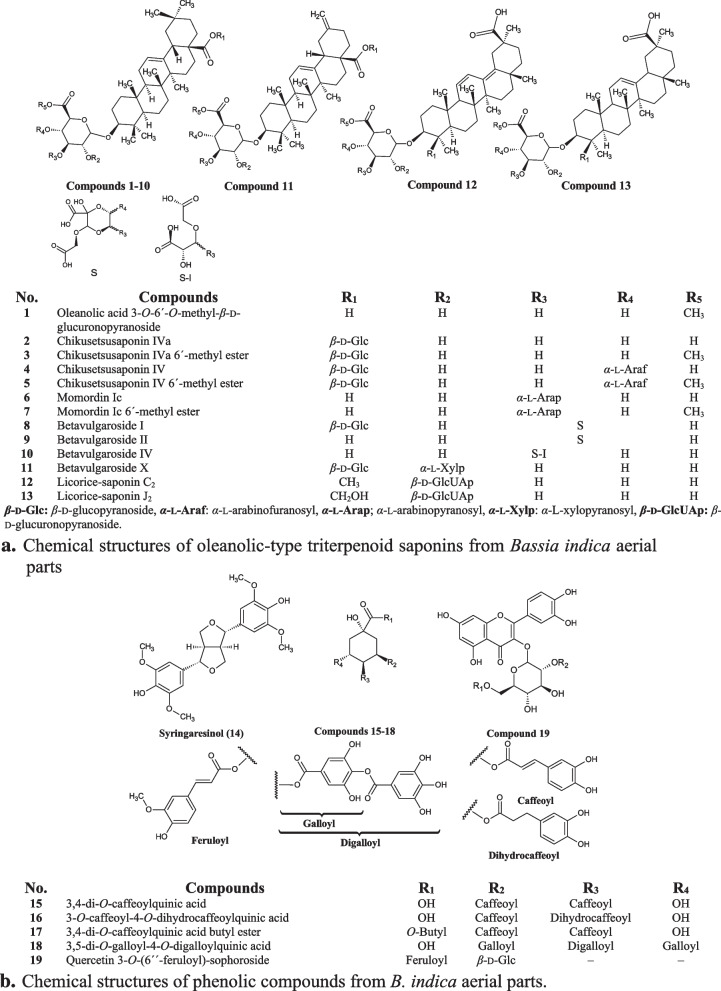
**a** Chemical structures of oleanolic-type triterpenoid saponins from *Bassia indica* aerial parts. *β*-D-Glc: *β*-D-glucopyranoside, *α*-L-Araf: *α*-L-arabinofuranosyl, *α*-L-Arap; *α*-L-arabinopyranosyl,* α*-L-Xylp: *α*-L-xylopyranosyl, *β*-D-GlcUAp: *β*-D-glucuronopyranoside. **b** Chemical structures of phenolic compounds from *B. indica* aerial parts

Molecular Operating Environment (MOE, 2019.0102) software was used to carry out the molecular docking studies. All minimizations were performed with MOE until a Root Mean Square Deviation (RMSD) gradient of 0.05 kcal mol^−1^ Å^−1^ with MMFF94x force field, and the partial charges were automatically calculated. The X-ray crystallographic structure of protein targets (PDB ID: 1OJ0 and 2AZ5) were downloaded from RCSB protein data bank (https://www.rcsb.org/). For molecular docking simulations, protonate 3D protocol in MOE with its default options was used to prepare the protein chains under study, by removing all residues and adding polar hydrogens, producing favorable protonation states for the simulations. The chemical structures of all identified compounds (Fig. 1a, b) were built using ACD/ChemSketch software, and they were converted into their canonical simplified molecular-input line-entry system (SMILE), then applied to MOE platform for energy minimization using the Merck molecular force field 94 (MMFF94x).

Hydrogens and partial charges were added, and conformational analysis of ligands were generated from a single 3D conformation by conducting a systematic search so that all combinations of dihedral angles were created for each ligand. Then, validation of the docking process was done by re-docking of the co-crystalized ligand with its receptor protein, and RMS (Root Mean Square) distance with MMFF94X force-field and the partial charges were automatically calculated. The co-crystalized ligand or site finder protocol (using MOE-Alpha Site Finder module) was used to define the active site for docking, *i.e.,* binding pocket, ligand interactions, and all amino acids in the active site, were established, recorded and isolated in the Dummy-form using ´site finder` module implemented in MOE, 2019.0102 [35].

Triangle Matcher placement method and London-dG scoring function were used for docking pose generation. A maximum 5 refinements out of 50 conformers (poses) were considered for each ligand. The selection of the best ligand conformation was based on; the interactions with the binding cavity target site's amino acids (No. of H-bonds, π-H, π-π, hydrophobic, etc.), the most favorable (least) free binding energy & distance and RMSD (≤ 2). The top scored poses of each cluster were considered for further calculations, with only the best pose is the one closest to the crystal structure that can be professed with sufficient certainty to occur in nature.

#### Prediction of the 3D structures of *Ts*-CF1 and *Ts*-CRT

The amino acid sequences of *T. spiralis* cathepsin F (*Ts*-CF1) and calreticulin (*Ts*-CRT) (GenBank: XM_003378197.1 and XP_003371379.1 respectively) were retrieved from the NCBI GenBank database on the FASTA format. Homology modeling of the mature domain of *Ts*-CF1was performed with Phyre2 based on the crystal structure of human cathepsin F (PDB ID: 1M6D) as a template, with 53% sequence similarity. Moreover, the 3D *T. spiralis* calreticulin structure was established based on the crystal structure of human calreticulin arm domains (PDB ID: 3RG0) as a template; with 53% sequence similarity. The resulted 3D structures of *Ts*-CF1 and *Ts*-CRT generated by Phyre2 were improved by energy minimized via YASARA force field (YASARA Energy Minimization Server), after which, the final models were submitted for structural analysis and verification server (SAVESv6.0—Structure Validation Server (ucla.edu)), which resulted with 87.2% & 88.7% of residues to be located in the most favorable geometric parts and 12.8% & 11.3% were in the additionally allowed parts of the Ramachandran plots, for *Ts*-CF1 and *Ts*-CRT respectively. Finally, the overall quality factors of 98.086 and 93.640 for *Ts*-CF1 and *Ts*-CRT respectively, confirmed that both models were modeled well within the range of a high-quality model [36].

#### In silico pharmacokinetics, toxicity predictions and drug-likeness properties

All the identified compounds (**1**–**19**) along with albendazole were converted into their canonical simplified molecular-input line-entry system (SMILE), then they were submitted to the SwissADME, PreADMET, ADMETlab 2.0 and Molsoft L.L.C. servers, in order to calculate their pharmacokinetic parameters including **A** (Absorption *i.e.,* Human intestinal absorption (HIA), Caco-2, MDCK and Skin permeability), **D** (Distribution *i.e.,* Plasma protein binding (PPB), P-glycoprotein substrate, inhibitor and C_Blood_-C_Brain_ barrier penetration (BBB)), **M** (Metabolism *i.e.,* CYP family substrate/inhibitor), **E** (Excretion *i.e.,* Elimination half-life (t_*1/2*_)) and **T** (Toxicity *i.e.,* Ames mutagenesis and Carcinogenesis), along with the drug-likeness score [37].

## Results and discussion

### Characterization of the triterpenoidal saponins and the phenolics

LC–ESI–MS/MS was conducted as the best choice for the identification of compounds in complex extracts with co-eluting peaks. LC/MS analysis was performed in -ve mode which produced a prominent deprotonated molecular ion [M–H]^–^ with weaker fragment ions. Whereas, the most abundant fragment ions corresponded to cleavage of the glycosidic bond accompanied by transfer of a hydrogen atom from the leaving sugar were resulted from + ve mode [38].

A total of **19** compounds (Fig. 1a, b) were identified based on the comparison of their pseudo-molecular ions and mass fragmentation patterns with the mass spectral data from the literature and compounds libraries.

The characterized compounds include **13** triterpenoidal saponins (Fig. 1a) of the oleanane-type base skeleton and were identified as; oleanolic acid 3-*O*-6´-*O*-methyl-*β*-D-glucuronopyranoside (**1**), chikusetsusaponin-IVa (**2**) and its methyl ester (**3**), chikusetsusaponin IV (**4**) and its methyl ester (**5**), momordin-Ic (**6**) and its methyl ester (**7**), betavulgaroside-I (**8**), -II (**9**) -IV (**10**), -X (**11**), licorice-saponin-C_2_ (**12**) and -J_2_ (**13**). In addition, **6** more phenolics (Fig. 1b) were identified as syringaresinol (**14**), 3,4-di-*O*-caffeoylquinic acid (**15**), 3-*O*-caffeoyl-4-*O*-dihydrocaffeoylquinic acid (**16**), 3,4-di-*O*-caffeoylquinic acid butyl ester (**17**), 3,5-di-*O*-galloyl-4-*O*-digalloylquinic acid (**18**) and quercetin 3-*O*-(6´´-feruloyl)-sophoroside (**19**). 

As appeared presented in (Table 1), compounds (**1**–**4**, **6**, **8**–**11**, **13**, **15**–**19**) produced at the same retention time both the protonated [M + H]^+^ and sodiated [M + Na]^+^ adduct in the + ve mode along with the deprotonated ion [M–H]^–^ in the –ve mode, which made it easy to confirm the molecular masses of these compounds, in addition, all compounds recorded an acceptable deviation (≤ 1 ppm) between their experimental and accurate masses. Moreover, compounds (**5**) and (**7**) are the methylated derivatives of (**4**) and (**6**) respectively, compound (**13**) is the hydroxy-methylene derivative of compound (**12**). Briefly, CID of compounds (**1–13**) showed the presence of fragment ions around *m/z* 439 and 425 corresponding to the aglycone moiety oleanolic acid (OA) with the loss of OH and/or OCH_3_ respectively, other fragment ions were generated upon the loss of GlcUAp at *m/z* 193, Glc at *m/z* 179, Xylp at *m/z* 149. Furthermore, compounds (**15**–**17**) produced the main characteristic fragment ions including loss of one or two caffeoyl moieties at *m/z* 179 characteristic for chlorogenic acid derivatives. To the best or our knowledge, compounds (**1**–**3**, **5**, **8**–**11**) were reported from *K. indica* [39], compounds (**4**, **6**, **7**) were reported from *K*. *scoparia* [40] and compounds (**14** and **19**) were isolated before from *B. indica* [15] and *B*. muricata [41, 42] respectively. In addition, compounds (**12**, **13, 18**) and (**15**–**17**) are reported in our study from *B. indica* for the first time [42, 43].

**Table 1:** LC–MS/MS of all identified compounds from *B. indica* aerial parts *n*-BuOH frac

**No**	**MF /MWt**	**R**_***t***_	**HRMS**	CID (*m/z*)	
				** + ve**	**–ve**
**1**	C_37_H_58_O_9_ *m/z* 646	10.51	647.41535	**647**[M + H]^+^, 669[M + Na]^+^, 453(M–H–GlcUAp)^+^, 425(M–CO–H–GlcUAp)^+^	645[M–H]^–^, 631[M–15]^–^
**2**	C_42_H_66_O_14_ *m/z* 794	7.98	795.45253	**795**[M + H]^+^, 817[M + Na]^+^, 633(M + H–Glc)^+^, 439(OA–OH)^+^, 421(OA–2H_2_O)^+^	793[M–H]^–^
**3**	C_43_H_68_O_14_ *m/z* 808	8.10	809.46818	**809**[M + H]^+^, 831[M + Na]^+^, 629(M-Glc)^+^, 425(M + 3H–179–207)^+^, 407(M–179–207–15)^+^	807[M–H]^–^
**4**	C_47_H_74_O_18_ *m/z* 926	9.96	927.49476	**927**[M + H]^+^, 949[M + Na]^+^, 439(M–Glc–GlcUAp–Xyl)^+^, 439 (OA–OH)^+^	925[M–H]^–^
**5**	C_48_H_76_O_18_ *m/z* 940	11.37	939.49479	–	**939**[M–H]^−^, 864(M–75)^–^, 761(M–H–179)^–^, 492(M–H–OA–2H_2_O)^–^, 178(Glc)^–^
**6**	C_41_H_64_O_13_ *m/z* 764	13.51	765.44196	**765**[M + H]^+^, 787[M + Na]^+^, 439(M–Glc–Xyl), 439(OA–OH)^+^	763[M–H]^–^
**7**	C_42_H_66_O_13_ *m/z* 778	9.02	777.44196	–	**777**[M–H]^–^
**8**	C_47_H_70_O_20_ *m/z* 954	9.63	955.45332	**955**[M + H]^+^, 977[M + Na]^+^, 747(M–Glc-CO), 439(M–Glc–S + OH)^+^, 439(OA–OH)^+^, 393(M–Glc–GlcUAp–S–CO)^+^, 247(S + CO_2_)^+^	953[M–H]^–^ 925(M–H–CO_2_)^–^
**9**	C_41_H_60_O_15_ *m/z* 792	11.19	793.40049	**793**[M + H]^+^, 815[M + Na]^+^, 653(M + Na-S)^+^, 455(M–GlcUAp–S)^+^, 437(M–GlcUAp–S–H_2_O)^+^	791[M–H]^–^, 629(M–H–S)^–^
**10**	C_41_H_62_O_15_ *m/z* 794	9.82	795.41615	**795**[M + H]^+^, 817[M + Na]^+^, 439[OA-OH]^+^, 393(OA–OH–COOH)^+^, 191(GlcUAp–2H)^+^	793[M–H]^–^
**11**	C_46_H_70_O_18_ *m/z* 910	9.17	911.46349	**911**[M + H]^+^, 933[M + Na]^+^, 749(M–Glc + H_2_O)^+^, 453(M–Glc–GlcUAp–Xylp + 31)^+^, 423(M–Glc–GlcUAp–Xylp + OH)^+^	909[M–H]^–^
**12**	C_42_H_62_O_15_ *m/z* 806	12.38	805.40049	–	**805**[M–H]^–^
**13**	C_42_H_64_O_16_ *m/z* 824	8.88	825.42671	**825**[M + H]^+^, 847[M + Na]^+^, 781(M-CO_2_)^+^, 423(M–2GlcUAp–CH_2_OH)^+^	823[M–H]^–^
**14**	C_22_H_26_O_8_ *m/z* 418	27.12	419.17004	**419**[M + H]^+^, 441[M + Na]^+^ 387(M–OCH_3_)^+^, 275(M–DMPh + 2H)^+^, 153(DMPh)^+^	–
**15**	C_25_H_24_O_12_ *m/z* 516	6.59	517.13405	**517**[M + H]^+^, 539[M + Na]^+^ 499(M–OH)^+^, 337(M–caffeoyl)^+^, 291(M–caffeoyl–CO_2_)^+^, 163(caffeic acid–OH)^+^	515[M–H]^–^
**16**	C_25_H_26_O_12_ *m/z* 518	6.73	519.14970	**519**[M + H]^+^, 541[M + Na]^+^ 357(M + H–caffeoyl)^+^, 163(caffeic acid–OH)^+^	517[M–H]^–^
**17**	C_29_H_32_O_12_ *m/z* 572	14.89	573.19665	**573**[M + H]^+^, 555(M–OH)^+^, 433(M–VHPh–OH)^+^, 335(M–Bu–caffeoyl)^+^, 256(M + H–VHPh–caffeoyl)^+^	571[M–H]^–^
**18**	C_35_H_28_O_22_ *m/z* 800	12.12	801.11449	**801**[M + H]^+^, 823[M + Na]^+^, 705(4 G + CO_2_H)^+^, 321(2 G)^+^, 185(G + OH)^+^	799[M–H]^–^
**19**	C_37_H_38_O_20_ *m/z* 802	24.82	803.20291	**803**[M + H]^+^, 430(M–Glc–feruloyl)^+^	801[M–H]^–^

*OA* Oleanolic acid (*m/z* 456), *Glc* Glucopyranosyl (*m/z* 179), *GlcUAp* Glucuronopyranosyl (*m/z* 193), *Me*GlcUAp Methyl glucuronopyranosyl (*m/z* 207), *Xylp* Xylopyranosyl (*m/z* 149), S: (see Fig. 1a), *DMPh* Dimethoxyphenol, *VHPh* vinyl-hydroxy phenol, *Bu* Butyl, *G* Galloyl

### In vitro Anthelmintic activity

The developmental stages of *T. spiralis* (adult, migratory and encysted) occur in the same host, hence it has been used as an interesting experimental model to assess the effectiveness of many anthelminthic agents [44].

### SEM findings of adult* T. spiralis*

**Fig. 2:**
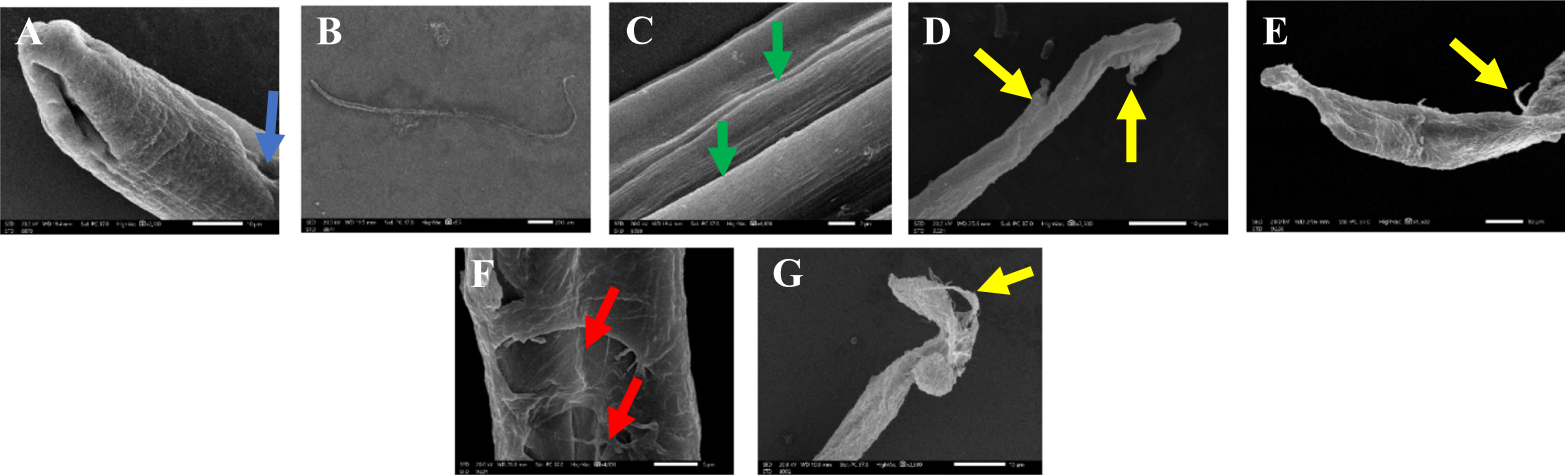
Scanning electron microscope findings of the cultured *T. spiralis* adult worm. **A** normal control group showing adult worm with intact annulated cuticle and openings of hypodermal glands, **B** infected control group showing normal adult worm cuticle with tapering end, **C** normal control group showing intact adult worm cuticle with normal longitudinal striation (green arrows), **D** and **E** *B. indica*-groups showing shrunked *T. spiralis* adult, sloughing of some areas of the cuticle (yellow arrow) and loss of the normal annulations in the cuticle, **F** *B. indica*-group showing marked destruction of the adult worm cuticle, sloughing of some areas of the cuticle (red arrows), **G** albendazole-group showing destruction of the adult worms and sloughing (yellow arrows) with loss of annulations

For infected control group cultured in an incubation medium only, the cuticle of the adult worm teguments retained the normal structure in the form of ridges, transverse creases and annulations, along with the appearance of openings of the hypodermal gland. Whereas, in *B. indica* BuOH frac. treated-groups, the adult worms showed shrinking, sloughing of some areas of the cuticle, loss of the normal annulations of the cuticle and marked destruction of the adult worm cuticle, besides, the albendazole-group showed destruction of the adult worms and sloughing with loss of annulations (Fig. 2).

### SEM findings of* T. spiralis* larval stage

**Fig. 3:**
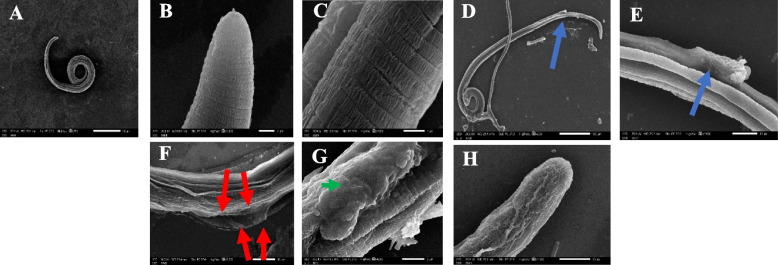
Scanning electron microscope findings of the cultured *T. spiralis* larvae. **A** infected control group showing normal comma shaped larva with intact cuticle and coiled posterior end. **B** and **C** normal control group showing larva with intact cuticular folds, transverse striations and shallow longitudinal grooves, **D**, **E**, and **F** *B. indica* BuOH frac groups showing *T. spiralis* larva with destructed shrunked cuticle, loss of its normal annulations and larval cuticle showing large blebs (blue and red arrows), **G** and **H** albendazole group showing large blebs (green arrow), and loss of the normal cuticular folds and striations

The infected control-group revealed coiled posterior with intact cuticular folds, transverse striations and shallow longitudinal grooves. Whilst, *B. indica* BuOH frac. group showed shrunken in *T. spiralis* larva, destructed cuticle with large blebs and loss of the normal annulations in the cuticle, aside from, albendazole group revealed large blebs with loss of the normal cuticular folds and striations (Fig. 3).

The shape of the parasite, nutrition and protection are dependent on cuticle integrity, which is an essential part of *Trichinella*’s body wall, along with the hypodermis and the somatic musculature it is necessary for osmoregulation [45]. Based on Thompson and Geary, who reported the principal mechanism of drug entry into the helminths to be a transcuticular passive diffusion, so anti-anthelmintics targeted the destruction of the worm’s surface [46, 47]. Which faced by the parasite’s blebbing as an attempt to replace damaged surface membrane in response to drug action. Therefore, the tegumental changes can be considered as a good indicator for the possible anthelmintic activity of a drug [48]. In our study, the electron microscopy scans showed severe destruction of the adult worm and larvae, marked cuticle swelling, areas with vesicles, blebs and loss of annulations in both *B. indica* BuOH frac. and albendazole treated-groups, while it retained its normal morphology when incubated in the culture medium only.

Anthelmintic drugs (**BzC**) can disrupt the parasite metabolism or destruct its cuticle/cytoskeleton which is finally lead to paralysis and eventual death [49]. Hence, the efficient antiparasitic activity of *B. indica* BuOH frac., could be mainly attributed to its chemical constituents (**1–19**) of saponins [13–15] and phenolics [19]. Briefly, the biological potential of saponins are basically due to their ability to cause changes in cell permeability via their specific interaction with the cell membrane [50]. Cavalcanti Gomes et al., hypothesized that saponins may be able to cause larval death via their ability to interfere with enzymatic pathways involved in larval development [51]. However, large concentrations of saponins are potentially toxic. Anyhow, in moderate concentrations, they can reduce the parasitic burden [52]. Moreover, Ekeanyanwu and Etienjirhevwe proved that phenolics can interfere with the energy generated by helminth parasites through the uncoupling of specific reductase-mediated reactions [53]. In addition, the high content of flavonoids, phenolic acids and tannins are correlated with high antioxidant property, which is in turn exhibited anthelmintic potential by interposing with energy generation in helminth parasites, uncoupling oxidative phosphorylation, and binding to free proteins in the gastrointestinal tract of the host animal and/or glycoprotein on the cuticle of the parasite and leading to death [54].

### In vivo results

**Fig. 4:**
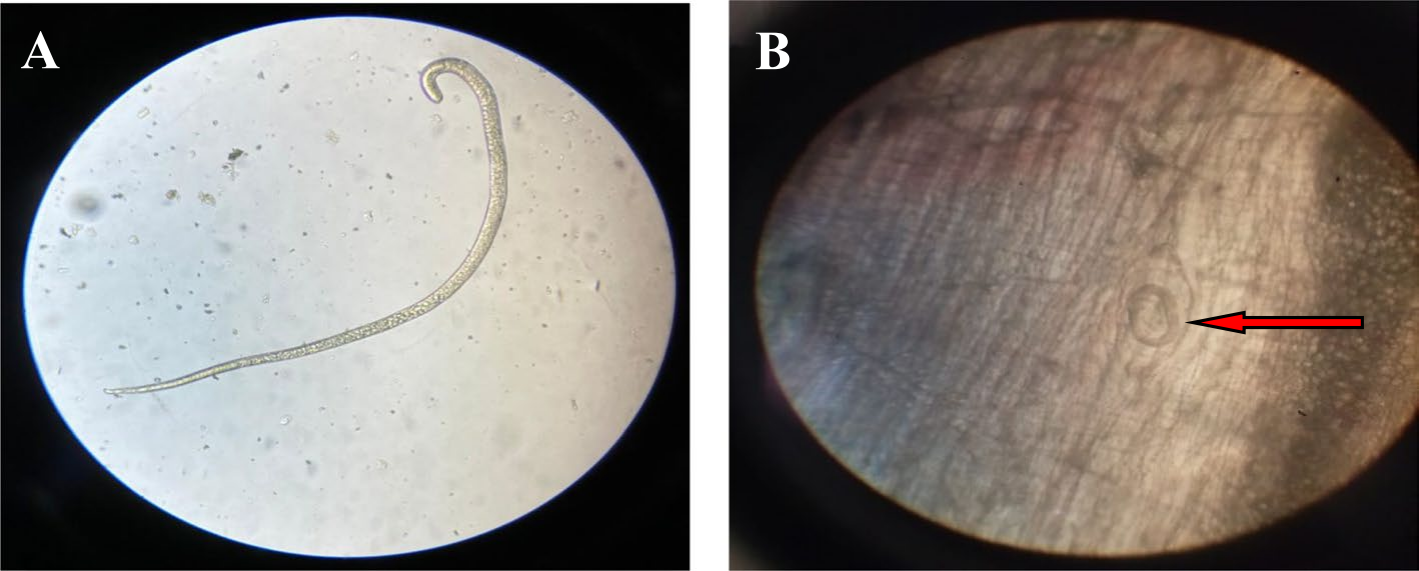
Photograph Represents *T. spiralis*. **a** Adult in the intestinal fluid on day 5 post infection (X 10). **b** Larvae in the diaphragm of mice on day 35 post infection (X 40)

To validate the potential in vitro outcomes, an in vivo study was conducted to assist the anti-parasitic activity of *B. indica* BuOH frac. compared with albendazole, during the (a)-intestinal phase (3–5 days p.i.), (b)-muscular phase (30–32 days p.i.) and (c)-intestinal then muscular phases (3–5 days p.i. and 30–32 days p.i.) separately (Fig. 4a, b).

### Adult worm count in the small intestine

As appeared in (Table 2), prophylactic treatment of the infected mice with *B. indica* BuOH frac. resulted in a significant reduction (*P* < 0.05) in the mean adult worm count (51 ± 8.4) with efficacy of 42.7%, compared to the control infected untreated group (**G-III**) with (89 ± 8.5). Moreover, a significant decrease in the mean number of adult worms (46.5 ± 3.5), with a drug efficacy of 47.8% was obtained in *B. indica* BuOH frac., treated group (**G-V**_**a**_) with (*P* < 0.01) in comparison to the control infected untreated group.

**Table 2:** The counts of *T. spiralis* adult worm and encysted larvae per gram muscle

***T. spiralis *phases**	**Mean ± SD**	***P*-value**	**% Reduction**
**Intestinal Phase (Day 6 post-infection)**			
**G-III**	89 ± 8.5		
**G-IV**	51 ± 8.4	< 0.05*	42.7
**G-V**_**a**_	46.5 ± 3.5	< 0.01**	47.8
**G-VI**_**a**_	7 ± 1.4	< 0.001***	92.1
**Muscular Phase (Day 35 post-infection)**			
**G-III**	7920 ± 973.1		
**G-IV**	1525 ± 330.4	< 0.001***	80.7
**G-V**_**b**_	2166.7 ± 351.2	< 0.001***	72.7
**G-V**_**c**_	1600 ± 556.8	< 0.001***	79.8
**G-VI**_**b**_	700 ± 173.2	< 0.001***	91.2
**G-VI**_**c**_	600 ± 100	< 0.001***	92.4

*P*-value is significantly different comparing with control, depending on Student t-test. *Initial *P*-value < 0.05 is significant. **Initial *P*-value < 0.01 is highly significant. **G-I**: negative control, **G-III**: Positive control (infected untreated), **G-IV**: prophylactically treated with *B. indica* BuOH frac., for seven days before infection, **G-V**: infected and treated with *B. indica* BuOH frac., **G-VI**: Infected and treated with albendazole

### Encysted larvae count in muscles

Prophylactic treatment of the infected mice by *B. indica* BuOH frac. significantly reduced (*P* < 0.001) the mean larval count per gram muscle (1525 ± 330.4) with an efficacy of 80.7% compared to the control infected untreated group (7920 ± 973.1). A significant decrease in the mean larval count per gram muscle was detected in the treated groups (*P* < 0.001) compared to the control infected untreated group. Moreover, better larvae eradications were found in groups that received two doses than groups received a single dose. Furthermore, the best reduction of the mean larval count (1600 ± 556.8) in the tested extract was found in **G-V**_**c**_ with efficacy of (79.8%). A significant reduction of the mean larval count (2166.7 ± 351.2) was observed in **G-V**_**b**_ with an efficacy of 72.7% for the single-dose regimen (Table 2).

In the present study, single-drug treatment significantly decreased the count of *T. spiralis*, but better larvae eradications were found in groups that received the drugs in two doses regimen (during intestinal and muscular phases). In the same context, Lu and co-workers demonstrated the in vivo anthelmintic activity of the MeOH extract of *K. scoparia* against *Dactylogyrus* intermedius (Monogenea) in goldfish (*Carassius auratus*) [55]. Moreover, Javed and colleagues reported the antifungal activity of *n*-BuOH frac. of the MeOH leaf extract of *K. indica* against *Macrophomina phaseolina* [22]. In addition, El-Wakil and co-workers evaluated the anti-trichinellosis activity of *Annona muricata* leaves ext. and stated that larval eradication was better with biphasic treatment over intestinal phase only [56].

### Histopathological results

#### Small intestine changes

**Fig. 5:**
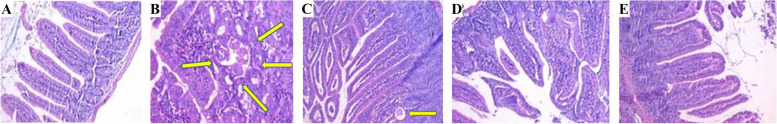
Sections in the intestine showing;  **A** Preserved villous pattern_ G-I. **B** Some *Trichinella* cysts and distorted villous pattern_G-III. **C** Some *Trichinella* cysts with mostly preserved villous pattern_G-IV. **D** Mostly preserved villous pattern_G-V_a_. **E** Absence of *Trichinella* cysts and preserved villous pattern_G-VI_a_. a, c, d, e; (H&E stain, X200) and b; (H&E stain, X400)

Histopathological examination of sections from the small intestine of the control negative (**G-I**) revealed a preserved villous pattern (Fig. 5a). Whereas, other sections of the infected control (**G-III**) showed dense intervillous inflammatory cellular infiltration consisting of mononuclear cellular infiltrate in the form of lymphocytes and plasma cells, there were broadening and atrophy of the intestinal villi with crypt hyperplasia with fragments of the adult worms were detected within the intestinal lumen (Fig. 5b). Sections examined from (**G-IV**) received *B. indica* BuOH frac. for seven days before infection, showed some *Trichinella* cyst with mostly preserved villous (Fig. 5c). However, sections examined from the treated group showed an obvious reduction in the intensity of the inflammatory cellular infiltration, with remarkable improvement of the other histopathological changes of the intestine, with a returning of the normal villous pattern (**G-V**_**a**_) with mostly preserved villous pattern (Fig. 5d). Furthermore, sections in the intestine of albendazole treated mice (**G-VI**_**a**_) showed the absence of *Trichinella* cysts and preserved villous pattern (Fig. 5e).

#### Skeletal muscle changes

**Fig. 6:**
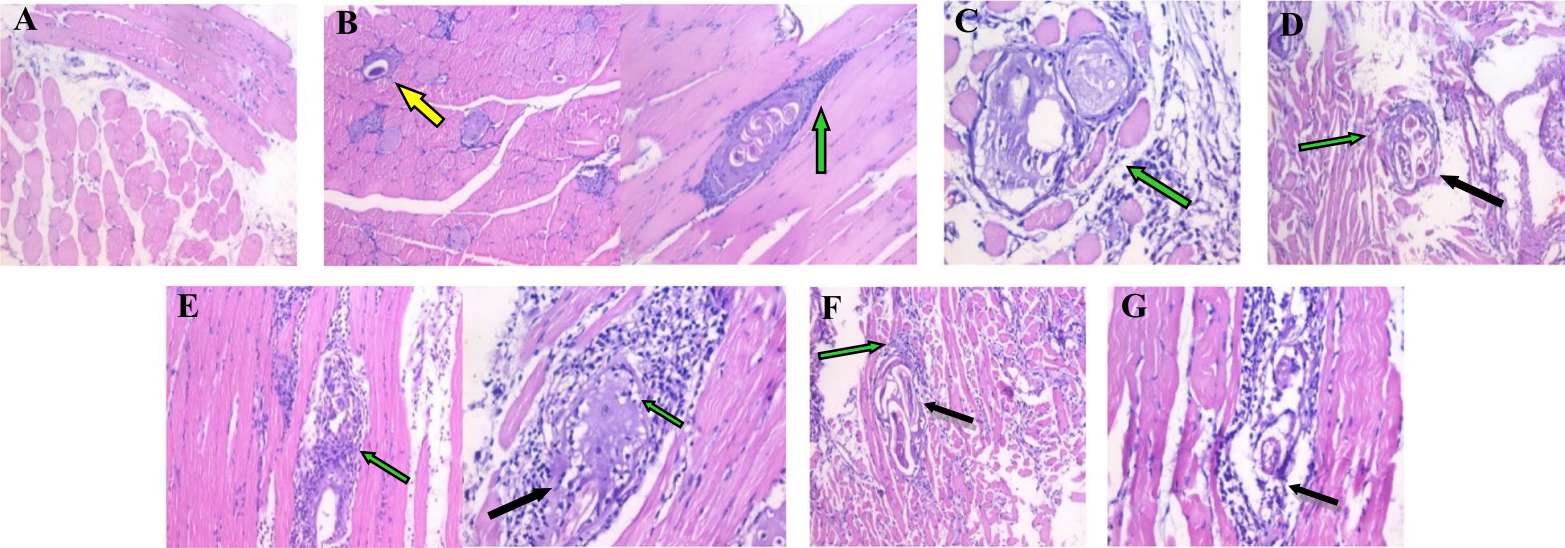
Histopathological muscular sections showing; **A** Non-infected and non-treated (control negative)_G-I. **B** Intact capsules (yellow arrow) and pericapsular plasma-lymphocytic inflammatory cellular infiltration (green arrow)_G-III. **C** Degenerated trichina capsule and larva and dense pericapsular plasma-lymphocytic inflammatory cellular infiltration (green arrow)_G-IV. **D** Degenerated capsule (black arrow) and focal pericapsular plasma-lymphocytic inflammatory cellular infiltration (green arrow)_G-V_b_. **E** Markedly degenerated capsule (black arrow) and larva with invasion by many macrophages (green arrow)_G-V_c_. **F** Focally degenerated capsule (black arrow) and mild pericapsular plasma-lymphocytic inflammatory cellular infiltration (green arrow)_G-VI_b_. **G** Degenerated trichina capsule (black arrow) and dense pericapsular plasma-lymphocytic inflammatory cellular infiltration (green arrow)_G-VI_c_. **a**, **b**, **d**, **f**: (H&E stain X200) and **c**, **e**, **g**: (H&E stain X400)

Histopathological examination of muscular sections from the infected control group (**G-III**) revealed the presence of a massive number of encysted *T. spiralis* larvae, diffusely in the sarcoplasm of the muscles and several chronic inflammatory cells in the form of lymphocytes, plasma cells, histiocytes infiltrating muscle bundles and surrounding the encysted larvae (Fig. 6b) compared to the control negative (Fig. 6a). Furthermore, the muscles from the prophylaxis group (**G-IV**) showed a focally degenerated trichina capsule and pericapsular histio-lymphocytic inflammatory cellular infiltration (Fig. 6c). Concerning the histopathological examination of muscular sections from mice groups that received the *B. indica* BuOH frac. for a single dose in the muscular phase only (**G-V**_**b**_), there were fewer trichina capsules with focally degenerated capsule and dense pericapsular plasma-lymphocytic inflammatory cellular infiltration (Fig. 6d). In addition, muscular sections from groups that received the *B. indica* BuOH frac. for two doses showed marked improvement of the histopathological finding compared to the infected control (Fig. 6e). Furthermore, muscular sections of albendazole treated mice showed focally degenerated capsule with mild to dense pericapsular plasma-lymphocytic inflammatory cellular infiltration (Fig. 6f, g).

Hence, histopathological examination of sections from the small intestine of the infected control group showed dense intervillous inflammatory cellular infiltration, with broadening and atrophy of the intestinal villi with crypt hyperplasia. Moreover, fragments of the adult worms were detected within the intestinal lumen. Muscular sections from the infected control group revealed the presence of a massive number of encysted *T. spiralis* larvae diffusely present in muscle sarcoplasm and a number of chronic inflammatory cells. In addition, the reduction of these destructive and inflammatory changes was evident in the treated groups. Groups with the combination therapy exhibited the best improvement in restoring the normal architecture, the presence of the least number of trichina capsule with degenerated capsule and focal pericapsular plasma-lymphocytic inflammatory cellular infiltration [57].

#### Immunostaining for TNF-α (a-proinflammatory cytokines)

**Fig. 7:**
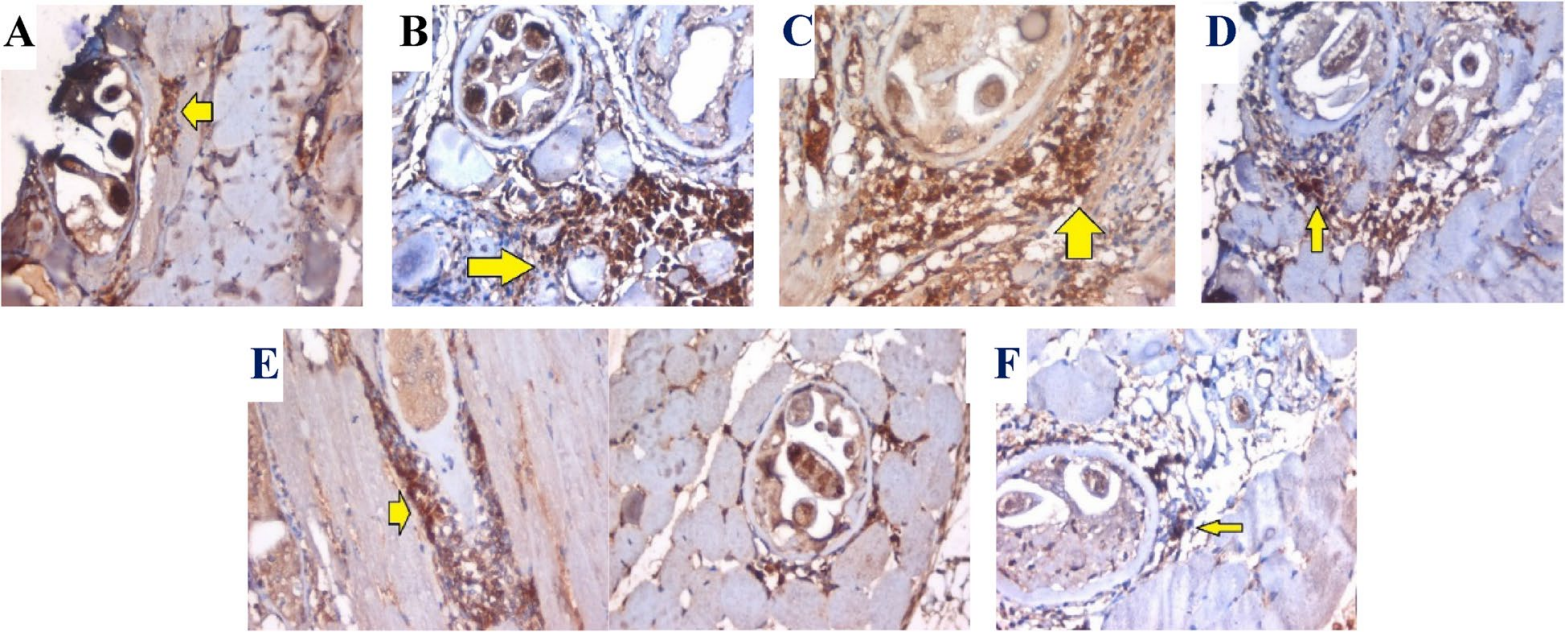
Sections in skeletal muscle showing *T. spiralis* trichina capsules (IHC, TNF-*α*, DAB, X400). **A** With focal capsular degeneration and degenerated contents, surrounded by some mono and polymorphnuclear inflammatory cells exhibiting moderate expression of TNF-*α* (arrow)_G-III control positive (infected untreated). **B** Surrounded by large number of mononuclear inflammatory cells exhibiting marked expression of TNFα (arrow)_G-IV (prophylactic). **C** Surrounded by large number of mono and polymorphnuclear inflammatory cells exhibiting marked expression of TNF-*α* (arrow)_G-V_b_. **D** Surrounded by large number of mono and polymorphnuclear inflammatory cells exhibiting only focal expression of TNF-*α* (arrow)_G-V_c_. **E** Surrounded by moderate number of mono and polymorphnuclear inflammatory cells exhibiting high percentage cellular expression of TNF-*α* (arrow)_G-VI_b_. **F** Surrounded by small number of mono and polymorphnuclear inflammatory cells exhibiting mild expression of TNF-*α* (arrow)_G-VI_c_

Section in skeletal muscle of the infected untreated control positive (**G-III)** showed trichina capsules with focal capsular degeneration and degenerated contents, surrounded by some mono- and polymorphnuclear inflammatory cells exhibiting moderate expression of TNF-*α* (Fig. 7a). While, section in skeletal muscle of *B. indica* prophylaxis (**G-IV**) showed trichina capsules surrounded by large number of mononuclear inflammatory cells exhibiting marked expression of TNF-*α* (Fig. 7b). However, section in skeletal muscle of (**G-V**_**b**_) received *B. indica* BuOH frac. in muscular phase only showed trichina capsules surrounded by large number of mono and polymorphnuclear inflammatory cells exhibiting marked expression of TNF-*α* (Fig. 7c). Moreover, in (**G-V**_**c**_) that received the drug in both intestinal and muscular phases, showed trichina capsules surrounded by large number of mono and polymorph nuclear inflammatory cells, and exhibited only focal expression of TNF-*α* (Fig. 7d). Besides, a moderate to mild expression of TNF-*α* was detected in (**G-VI**_**b**_ and **-VI**_**c**_), (Fig. 7e & 7f) respectively. Hence, it can be concluded that *B. indica* BuOH frac. lowered the parasite burden in the intestine and muscle tissues, and alleviating muscular inflammatory reaction, which is in a good agreement with the evidences indicated that inflammatory mediators are involved in the muscular pathogenesis of *T. spiralis* infection and accelerate the development and progression of myositis associated with this phase [58].

Monokines, such as IL-1, -6, -8, TNF-*α* and G-CSF, are immune molecules that are secreted mainly by monocyte and macrophages, and play a vital role in mediating and regulating immune and inflammatory reactions as reflected by proinflammatory factor, which perform its biological function in a pleiotropic way on multiple cell types and play a pivotal role in the pathogenesis of chronic inflammatory diseases [59]. Our results proved the anti-inflammatory activity of that *B. indica* BuOH frac. in *T. spiralis* infection via depressing the expression of TNF-*α* in the muscle tissues. However, the experimental results clearly demonstrated that both *B. indica* BuOH frac. and albendazole were effective against intestinal adult worms and muscle larvae. In addition, TNF-*α* was controlled at low levels due to the bioactivity of *B. indica* BuOH frac., which indicated its powerful anti-inflammatory function in *T. spiralis* disease. These results are in consistent with Xu and co-workers who reported that *Vaccaria* *n*-BuOH extract lowered the production of proinflammatory cytokines as TNF-*α* and the infection burden of *T. spiralis* in vivo [60]. Therefore, *B. indica* BuOH frac. may achieved anti-inflammatory effect by relieving the inflammatory response of the infected mice and eventually achieved the nematicidal effect of *T. spiralis*. Notably, although albendazole plays a more effective role in reducing the burden of *T. spiralis* larvae, but due to its limitation in clinical use, *B. indica* BuOH frac. may be an ideal option to treat human trichinellosis. The resulted promising anti parasitic potential of *B. indica* BuOH frac. constituents arouse our interest to go further for an in silico molecular docking investigation of its identified compounds (**1–19**) as novel anti *T. spiralis*.

### Molecular docking results

Recently, drug discovery development expressed in the computational studies which involve the use of algorithms and programs, have led to a regenerated interest for predictions of therapeutic interventions in biological processes. Molecular docking approach, which foretells binding interactions between target receptors and ligands at the binding pocket active site, it represents an intrinsic technique that virtually checking several thousands of ligands against target receptors and identify their potential inhibitors with high accuracy and speed [61].

MOE (2019,0102) was conducted to perform a detailed in silico study of all identified compounds (**1–19**) from *B. indica* BuOH frac. with respect to selected protein targets (Table 3). Briefly, as reported that albendazole targets selectively *β*-tubulin monomer of the parasite and inhibits its microtubule polymerization [9, 10]. So, (PDB ID: 1OJ0) was the one of choice for this goal, all the docked compounds (**1–19**) fit into the binding site with different binding affinities compared to the co-crystalized ligand (**ABZ**) with RMSD < 2 Å. Out of this list, only compounds (**14–17**) revealed closest binding energies to **ABZ**, while the others revealed a relatively high (+ ve) unfavorable binding energies, which could be attributed to their high molecular weights (418–954 Da) compared to that of **ABZ** (265 Da) and its active pocket. However, their binding energies may be improved via multipose binding in the docking process [62].

**Table 3:** Docking scores and receptors amino acids involved in the interactions with the ligand compounds

**Ligands**	**1OJ0**		**3D *Ts*-CrT_3RG0**		**3D *Ts*-CF1_1M6D**		**2AZ5**	
	∆G*	**Receptor Amino Acids Involved in Interactions and Distance (Å)**	∆G*	**Receptor Amino Acids Involved in Interactions and Distance (Å)**	∆G*	**Receptor Amino Acids Involved in Interactions and Distance (Å)**	∆G*	**Receptor Amino Acids Involved in Interactions and Distance (Å)**
**ABZ**	-7.67	**Val236** (3.38), **Ser165** (2.84), **Gln134** (3.27)	-5.01	Glu201^a^ (3.21), His302^c^ (3.59)	-5.22	Gly168^a^ (3.05), Trp335^c^ (3.55)	-5.74	**Tyr119**^**c**^ (4.33)
**1**	58.53	***Glu198***^***a***^ (3.01), **Val236**^**a**^ (2.78), Leu250^b^ (2.87)	-6.42	Thr207^a^ (2.93), Glu201^a^ (2.97) Arg162^b^ (3.11 & 3.05)	-7.00	**Cys173**^**a**^ (3.56), Asp166^a^ (3.49 & 2.97)	-7.61	**Tyr151**^**b**^ (3.07)
**2**	84.79	**Ser165**^**a, b**^ (2.52 & 3.06), Asn226^b^ (3.42), Cys12^b^ (3.23)	-7.23	**Asp202**^**a**^ (2.78), **Arg199**^**b**^ (2.98)	-7.32	Asp166^a^ (2.90 & 2.81), **Cys173**^**a**^ (3.38), Asn308^a^ (3.45 & 3.03), Trp335^c^ (4.35)	-9.70	**Tyr119**^c^ (3.60)
**3**	85.16	Val226^a^ (2.50), **Ser165**^**b**^ (3.14), Asn226^b^ (2.82), ***Phe200***^***c***^ (3.70)	-6.88	Glu201^a^ (3.30), **Asp202**^**a**^ (2.82 & 3.47), **Arg199**^**b**^ (3.20) **Lys163**^**b**^ (3.10)	-7.79	Asn308^a^ (3.35), **Cys173**^**a**^ (3.21) Asp166^a^ (2.90 & 2.95 & 3.05)	-8.43	Leu120^a^ (2.92), **Tyr151**^b^ (2.79)
**4**	66.66	Asn204^a^ (3.08), Asn226^a^ (3.09), ***Glu198***^***a***^ (2.73), **Ser165**^**b**^ (2.84), **Gln134**^**b**^ (2.61)	-7.96	Glu201^a^ (3.46), **Arg199**^**b**^ (3.12)	-7.82	Asn308^a^ (3.13), **Cys173**^**a**^ (3.04) Trp335^a^ (3.42), Asp166^a^ (2.89)	-9.10	**Tyr151**^**b**^ (2.99), **Tyr59**^**c**^ (4.25)
**5**	102.98	***Glu198***^***a***^ (2.82 & 3.29), **Val236**^**a**^ (3.35), **Ser165**^**a,b**^ (2.87 & 2.53), Tyr222^b^ (2.79)	-7.69	**Asp202**^**a**^ (3.55), Ser185^a^ (3.14) Asn183^b^ (3.39)	-8.34	Asn308^a^ (3.18), Glu212^a^ (2.98) **Cys173**^**a**^ (4.06), Asp166^a^ (3.00) Trp174^b^ (3.32), Trp335^c^ (4.30 & 3.99)	-9.97	**Tyr151**^**b**^ (2.72)
**6**	51.58	Met233^a^ (3.36), **Gln134**^**b**^ (2.50), ***Tyr50***^***b***^ (2.93), His28^b^ (3.26)	-6.74	–	-7.50	**Cys173**^**a**^ (3.16), Gly214^b^ (3.07)	-8.21	**Tyr119**^**b**^ (2.90)
**7**	78.49	***Glu198***^***a***^ (2.56 & 2.61), Met233^a^ (2.68), **Gln134**^**b**^ (2.61)	-6.85	Lys157^b^ (3.10)	-7.34	Asn308^a^ (3.09), **His309**^**a**^ (3.44) **Cys173**^**a**^ (3.68)	-7.94	**Tyr151**^**b**^ (3.02)
**8**	134.90	Met233^a^ (3.81), Ile202^a^ (3.30), Thr237^a^ (3.23), ***Glu198***^***a***^ (2.40 & 2.37), **Val236**^**a**^ (3.03), **Ser165**^**b**^ (2.52), Leu250^b^ (2.82)	-7.18	**Asp202**^**a**^ (3.48), **Glu187**^**a**^ (3.17) Glu201^a^ (2.85), **Arg199**^**b**^ (3.13) Leu210^b^ (3.35)	-7.87	**His309**^**a**^ (3.13)	-9.64	Gly121^a^ (2.95), **Tyr151**^**b**^ (2.93) **Tyr59**^**c**^ (4.48)
**9**	79.28	**Ser168**^**a**^ (3.15), Thr237^a^ (2.38), Phe20^c^ (4.11)	-6.68	**Asp202**^**a**^ (3.12 & 2.96)	-6.81	**Cys173**^**a**^ (3.49), Trp335^c^ (4.09 & 4.53)	-8.21	–
**10**	72.24	Met233^a^ (3.28), **Ser165**^**a**^ (3.19 & 2.39), **Gln134**^**b**^ (2.83), Thr237^b^ (2.93)	-7.35	**Arg199**^**b**^ (3.13)	-6.85	Ile294^a^ (2.93), His296^a^ (2.92)	-8.83	**Tyr59**^**c**^ (4.01)
**11**	95.55	***Phe167***^***a***^ (2.79), Met233^a^ (3.01), **Val236**^**a**^ (3.13), Thr237^a^ (2.41), ***Glu198***^***a***^ (2.64 & 2.45), Asn204^a^ (2.55 & 2.66), Leu250^b^ (3.08), **Ser165**^**b**^ (2.56), ***Phe167***^***c***^ (4.83)	-7.15	**Asp202**^**a**^ (3.41 & 2.86) **Glu187**^**a**^ (2.89), Thr207^a^ (2.88) Arg162^b^ (3.00), **Lys194**^**b**^ (3.05)	-8.15	Asn308^a^ (3.32 & 2.79), **Cys173**^**a**^ (3.24), Trp335^a,c^ (3.09 & 3.88), Asp166^a^ (2.81)	-9.03	**Tyr119**^**a**^ (3.04)
**12**	73.39	Met233^a, b^ (3.33 & 3.29), **Val236**^**a**^ (2.87), **Ser165**^**b**^ (2.91), **Gln134**^**b**^ (2.67), Thr238^b^ (2.67)	-6.85	Glu201^a^ (2.81), **Asp202**^**a**^ (2.90)	-7.51	Asp166^a^ (3.03), Asp166^a^ (3.16) Trp335^c^ (3.86)	-7.83	Leu157^a^ (2.92), Lys98^b^ (3.10 & 3.18), Lys11^b^ (3.21), **Tyr119**^**c**^ (3.96)
**13**	89.38	Met233^a^ (2.50), **Ser165**^**b**^ (2.36 & 2.77), Leu250^b^ (2.97), Thr238^b^ (2.74)	-7.22	Glu201^a^ (2.95)	-8.02	Asn308^a^ (3.23), **His309**^**c**^ (4.14)	-8.21	**Tyr119**^**a**^ (3.27)
**14**	0.44	Cys12^a^ (4.01), Met233^c^ (3.93), ***Phe167***^***d***^ (3.86)	-5.39	Leu210^b^ (3.17)	-6.13	**His309**^**a**^ (2.99), Trp335^d^ (3.77)	-6.98	**Leu57**^**c**^ (4.27), **Tyr119**^**e**^ (3.81)
**15**	6.07	**Val236**^**a**^ (2.93), ***Glu198***^***a***^ (2.64), **Gln134**^**a**^ (2.57), **Gln134**^**b**^ (2.57), Thr238^b^ (2.97)	-5.98	**Asp202**^**a**^ (3.47), Arg162^b^ (3.38)	-6.69	Asn169^a^ (2.92), Asp166^a^ (3.20) **Cys173**^**a**^ (3.37), Gly171^c^ (4.68) Trp335^d^ (3.88)	-7.77	**Tyr59**^**c**^ (4.22)
**16**	4.57	***Glu198***^***a***^ (2.74), Asp249^a^ (2.84), Leu250^a^ (3.21), **Ser165**^**b**^ (3.16)	-6.08	Thr207^a^ (2.78), Glu201^a^ (2.95 & 2.90), Leu210^b^ (3.08)	-6.76	**Cys173**^**a**^ (3.18), Gly213^c^ (3.80)	-7.58	**Tyr119**^**c**^ (4.07)
**17**	6.16	**Val236**^**a**^ (3.14), Glu27^a^ (3.03), **Ser165**^**a**^ (3.11), **Gln134**^**b**^ (2.80)	-6.07	**Asp202**^**a**^ (2.99), His211^a^ (3.00) Glu201^a^ (2.78), Leu210^b^ (3.02) Arg162^b^ (2.99), Thr205^c^ (3.82)	-6.93	**His309**^**a**^ (3.48)**,** Asp166^a^ (3.31) **Cys173**^**a**^ (3.59), Met288^a^ (3.03) Gln167^b^ (2.98)	-7.90	–
**18**	33.67	Ser230^a^ (3.19), ***Glu198***^***a***^ (2.51), **Val236**^**a**^ (2.99), **Gln134**^**b**^ (2.44 & 2.63), **Ser165**^**b**^ (2.95)	-7.00	**Asp202**^**a**^ (2.90), Thr207^a^ (2.95) His211^b^ (2.97 & 3.54)	-7.59	Asn169^a^ (3.11), **Cys173**^**a**^ (3.14) Asp166^a^ (2.88), Asn169^a^ (3.00) Trp335^c, d^ (4.57 & 3.80)	-8.53	**Tyr119**^**a, c**^ (2.94 & 4.69), Leu157^a^ (2.80), Lys11^b^ (3.04), **Tyr59**^**c**^ (4.45)
**19**	58.36	Met233^a^ (3.51), ***Phe167***^***a***^ (2.44), Thr237^a^ (2.38), Glu27^a^ (2.98), **Gln134**^**b**^ (2.78), **Ser165**^**b**^ (2.90)	-7.46	**Asp202**^**a**^ (3.40), Glu201^a^ (3.01) His302^a^ (3.12), Arg162^b^ (3.09) **Arg199**^**b**^ (2.97), Pro209^c^ (4.61) Leu210^c^ (4.67)	-7.75	Asp166^a^ (3.43), **Cys173**^**a**^ (3.60) Glu212^a^ (2.85), Gln167^b^ (2.97) **His309**^**c**^ (3.99), Asn308^c^ (4.59) Trp335^c^ (3.95)	-8.47	**Tyr151**^**b**^ (3.21)

^*^ (Kcal/mol), ^a^ H-donor, ^b^ H-acceptor, ^c^ H-Pi, ^d^ Pi-Pi, ^e^ Pi-cation

**Bold:** Key amino acid residues, ***Bold and italic:*** Key amino acid residues associated with resistance mutation

Nevertheless, the binding affinities reflect the potential inhibition of *B. indica* BuOH frac. constituents (**1–19**) against microtubule polymerization. However, the ligand size plays a vital role in docking process *i.e.,* larger ligands preserve a considerable docking challenge due to their higher flexibility, beside they are more susceptible to form higher number of interactions with the protein receptor. Thus, the binding energy of the resulted complexes may not be considered as a determinant property to clarify their activity against the parasite, and we can relate the inhibition of microtubule polymerization of the parasite to the interactions of all compounds (**1–19**) with the 3 key interacting residues amino acids (Val236, Ser165 and Gln134) of the active pocket [63].

**Fig. 8:**
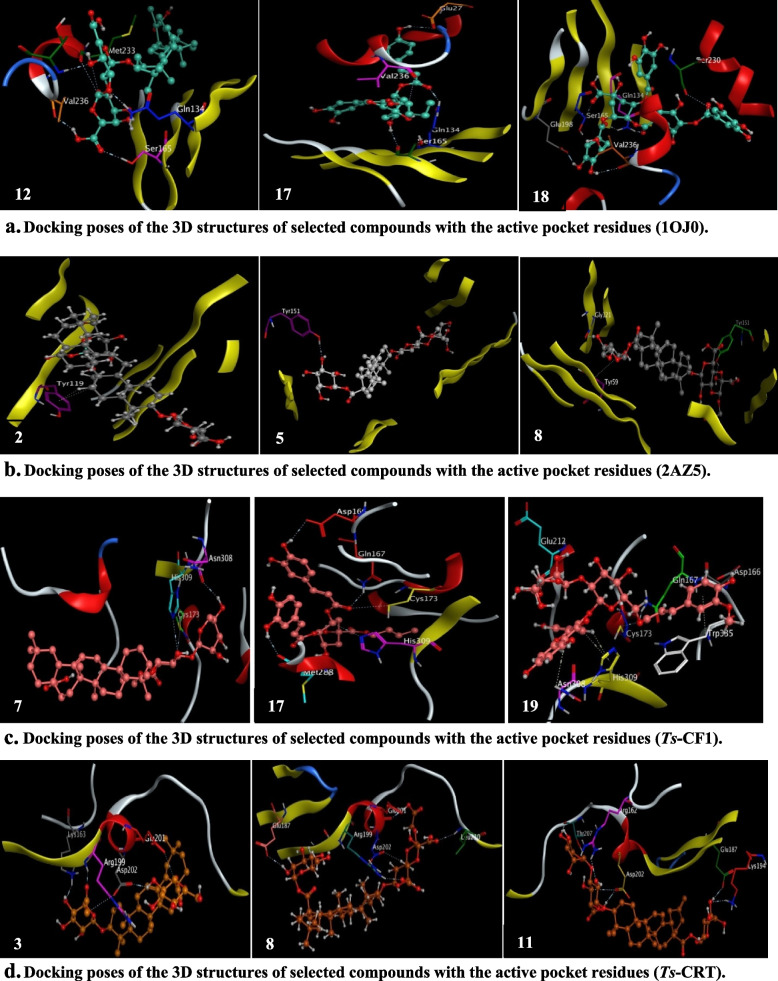
**a** Docking poses of the 3D structures of selected compounds with the active pocket residues (1OJ0). **b** Docking poses of the 3D structures of selected compounds with the active pocket residues (2AZ5). **c** Docking poses of the 3D structures of selected compounds with the active pocket residues (*Ts*-CF1). **d** Docking poses of the 3D structures of selected compounds with the active pocket residues (*Ts*-CRT)

Molecular docking of ligand molecules (**1–19**) with the protein receptor proved that all molecules bonded with one or other amino acids in the active pocket (Table 3), out of all frontrunner compounds (**1–19**), six compounds (**4, 5, 8, 11, 15, 19**) exhibited two amino acid interactions, three compounds (**12, 17, 18**) were found to have exploited the same binding pocket and interacted with the same 3 key amino acid residues within the receptor site (Fig. 8a), and only compound (**17**) revealed the most favorable and lowest binding energy among others. It was observed that all the frontrunner compounds exhibited one or more interactions with the key amino acids at distances much better than the co-crystallized ligand itself. In addition, Aguayo-Ortiz and co-workers, identified that (Phe167, Glu198 and Phe200) are the most important amino acids associated with the resistance mutations of helminths [64]. Moreover, Robinson and colleagues, affirmed that (Tyr50, Gln134, Thr165 and Met257) are the amino acids identified as **BzC** resistance mutations [65]. Hence, it can be documented from (Table 3) that compounds (**1**, **3–8**, **11**, **14–16**, **18**, **19**) out of the others interacted with one or more of amino acids associated with the resistance mutations, which strengthen their potential anti-anthelmintic.

As proved from the in vivo study that the activity of *B. indica* BuOH frac. may be attributed to its potential anti-inflammatory via depressing the expression of TNF-*α* in the muscle tissues, this encourage our group to explore the in-silico binding affinities of all identified compounds (**1–19**) from the BuOH frac. with tumor necrosis factor alpha (TNF-*α*). Zia and co-workers, asserted that Tyr119 is a crucial for TNF-*α* inhibition along with Leu57, Tyr59, Ser60, Gln61 and Tyr151 [66]. Molecular docking results of the ligand compounds (**1–19**) yielded complexes with binding energies ranged from (–9.97 to –6.98 kcal/mol) far less than that of **ABZ** (–5.74 kcal/mol), and even superior with less bonding distances < 4 Å. Out of the frontrunner list, **ABZ** and compounds (**2, 6, 11–14, 16, 18**) interacted with the pivotal amino acid Tyr119, in addition, compounds (**2, 5, 8**) represented the most energetical favorable ligands with outstanding binding energies (> –9.5 kcal/mol) and thus are speculated to have better binding affinities (Fig. 8b).

Tracking the life cycle of *T. spiralis* revealed that cathepsin F specific (*Ts-*CF1) is a cysteine protease existed in cuticle and stichosome, and it is an essential for their life stages, this is favoring its potential use as a drug target against *T. spiralis* infection [6]. Moreover, Shao and fellows, proved that *T. spiralis* lean to escape the host innate and adaptive immune attack via some sophisticated mechanisms [7], one of which is the secretion of calreticulin protein (*Ts*-CRT) on the surface of *T. spiralis* at different stages of adult and muscle larvae worms, which binds, interferes and overlaps with the host immune system to facilitate their survival, so targeting this protein receptor may help against their infection [67].

To unveil the effect of all identified compounds (**1–19**) against *T. spiralis* infection, a homology modeling protocol was performed to prepare the 3D structures of both *Ts*-CF1 and *Ts*-CRT, and were averred via their overall quality factors to be within the range of a high-quality model. The cysteine protease conserved active site of Cys173, His309 and Asn333 which were identified and revealed in the propeptide of *Ts*-CF1 [6]. While, in silico docking studies predicted that C1q binding sites in *Ts*-CRT are mainly located between Lys163 in the N-domain and Asn286 in the P-domain, with an active pocket composed of Lys163, Asn164, Glu187, Asp193, Lys194, Glu195, Tyr197, Arg199, Asp202, Trp223, Glu278, Trp279, Ala280, Glu282, Gln283 and Asn286 [7].

It is apparent clearly from (Table 3) that most of the compounds (**1–19**) had better binding affinities and inhibitory activities against *T. spiralis* infection compared to **ABZ**, with binding energies ranged between (–6.13 and –8.34) kcal/mol for the 3D_*Ts*-CF1/ligand complexes, and with (–5.39 and –7.69) kcal/mol for the 3D_*Ts*-CRT/ligand complexes, better than **ABZ** with (–5.22 and –5.01) kcal/mol respectively.

In silico docking of ligand molecules (**1–19**) with the target site revealed that all molecules displayed binding affinities with the key or the other amino acids in the active pockets (Table 3). For the 3D_*Ts*-CF1/ligand complexes, the key amino acids of the active pocket were targeted by all compounds (**1–19**) except compounds (**12**) and **ABZ**. Out of all, only compounds (**7**, **17**, **19**) exhibited molecular interactions with two active pocket residues (Fig. 8c). On the other hand, the 3D_*Ts*-CRT/ligand complexes revealed the interacted amino acids involved in the in silico active pocket, and clarify that compounds (**2–5, 7–19**) interacted with one or more of the active pocket residues, although compound (**6**) showed no interactions, but compounds (**3**, **8**, **11**) have the best interactions with three key amino acids (Fig. 8d).

The intermolecular interactions (HB-donor, HB-acceptor, H-π, π-π, π-cation) play a prime role in energetically stabilizing ligands at the binding site of protein receptors. Among all types of molecular interactions, hydrogen bonding represented the predominated type in our in-silico study, though other hydrophobic interactions were also participated, however, hydrogen bond networks assist the binding strength between target receptor and compounds (**1–19**), *e.g.,* hydrogen bonds modulate the ligand/receptor binding affinity which is usually a result of setting up of like-for-like synergistic interactions [68].

Our findings of the molecular docking are suggestive of *B. indica* BuOH frac. containing compounds (**1–19**) having the ability to affect the target enzyme systems in such a way and/or even better as the co-crystallized ligand, which is, an indicative for their potential anthelmintic activity against *T. spiralis* at different life stages.

### ADMET predictions

The activity of a certain drug target can be affected by certain pharmacokinetic factors *i.e.,* solubility, bioavailability and metabolism, as a score of enzyme induction or inhibition [69]. The prediction of absorption, distribution, metabolism and excretion (ADME) proprieties for all identified compounds (**1–19**) are shown in (Tables 4 and 5).

**Table 4:** Drug-likeness, oral bioavailability and absorption predictions of all compounds

**Comps**	**HBA**	**HBD**	**n-rotb**	**TPSA** **(Å**^**2**^**)**	**Log *P***_***o/w***_	**Log *s***	**Ro5**	**HIA** **%**	**P**_**MDCK**_	**P**_**Skin**_	**P**_**CaCo2**_
**ABZ**	4	2	6	52.53	2.80	-3.37	0	88.19	16.27	-4.14	49.96
**1**	9	4	5	111.65	4.50	-5.08	2	90.89	0.04	-2.94	20.74
**2**	14	8	7	183.97	3.17	-3.26	3	26.05	0.04	-2.02	19.40
**3**	14	7	8	176.73	3.52	-3.74	3	43.34	0.04	-2.13	19.55
**4**	17	10	9	232.57	2.07	-1.82	3	5.64	0.04	-2.71	19.59
**5**	18	9	9	225.34	2.59	-2.26	3	10.75	0.04	-2.90	19.71
**6**	13	7	6	166.15	3.78	-3.88	3	44.72	0.04	-1.10	20.60
**7**	13	6	7	158.92	4.13	-4.19	2	63.78	0.04	-2.10	20.74
**8**	17	9	10	251.20	1.61	-2.28	3	3.24	0.04	-1.19	19.55
**9**	15	6	8	186.12	3.66	-3.90	2	29.38	0.04	-1.21	20.47
**10**	15	7	11	264.81	3.05	-4.42	3	29.20	0.04	-1.20	20.84
**11**	17	10	8	231.23	1.43	-2.23	3	5.88	0.04	-3.03	19.37
**12**	15	8	7	193.69	4.06	-4.20	3	18.69	0.04	-1.44	20.80
**13**	16	9	8	210.80	2.69	-3.44	3	7.07	0.04	-1.65	20.70
**14**	8	2	6	95.84	1.74	-1.52	0	94.14	0.20	-4.07	39.31
**15**	12	7	9	164.18	1.54	-1.88	3	23.12	0.04	-2.85	19.53
**16**	12	7	10	164.18	1.07	-1.86	3	19.76	0.04	-2.88	19.53
**17**	12	6	13	156.81	3.54	-3.06	3	50.72	0.04	-2.53	18.28
**18**	22	13	13	302.76	-1.11	-0.80	3	0.05	0.04	-2.53	13.09
**19**	20	11	12	258.79	0.46	-1.80	3	2.71	0.04	-3.09	14.67

*ABZ Albendazole*, *HBA* Hydrogen bond acceptor ≤ 10, *HBD* Hydrogen bond donor ≤ 5, *n-rotb* no. of rotatable bond ≤ 10, *TPSA* Topological polar surface area ≤ 130Å^2^, *Log Po/w* octanol/water partition coefficient -0.7 – + 5.0, *Log s* aqueous solubility scale Insoluble < -10 < Poorly < -6 < Moderately < -4 < Soluble < -2 < Very Soluble < 0 < Highly Soluble; *Lipinski* number of violations of Lipinski’s rule of five ´max. is 4`, *HIA* Human Intestinal Absorption, *P*_*CaCO2*_: Cellular permeability, *P*_*MDCK*_ Cell permeability Maden Darby Canine Kidney, *P*_*Skin*_ Skin permeability

**Table 5:** Distribution, toxicity, half-life and drug-scores predictions for all compounds

**Comps**	**T**_**1/2**_ **(h)**	**BBB** **C**_**brain**_**/C**_**blood**_	**P-gp**	**CYP2D6**	**CYP3A4**	**PPB** **%**	**Ames**	**Carcino test**		**DLs**
								**Mouse**	**Rat**	
**ABZ**	0.805	0.65	No	No	No	96.60	Mutagenic	+	-	0.68
**1**	0.114	0.63	Yes	No	Yes	94.87	Non-mutagenic	+	+	0.71
**2**	0.503	0.05	Yes	No	Yes	89.39	Mutagenic	+	+	0.81
**3**	0.261	0.07	Yes	No	Yes	88.70	Non-mutagenic	+	+	0.67
**4**	0.624	0.03	Yes	No	Yes	71.57	Mutagenic	+	-	0.86
**5**	0.482	0.04	Yes	No	Yes	71.92	Non-mutagenic	+	-	0.71
**6**	0.503	0.09	Yes	No	Yes	91.22	Mutagenic	+	+	0.80
**7**	0.290	0.10	Yes	No	Yes	89.70	Mutagenic	+	+	0.81
**8**	0.774	0.12	Yes	No	Yes	78.45	Non-mutagenic	+	+	0.87
**9**	0.636	0.04	Yes	No	Yes	89.81	Non-mutagenic	+	+	0.88
**10**	0.775	0.05	Yes	No	No	86.60	Mutagenic	+	+	1.11
**11**	0.705	0.03	Yes	No	Yes	65.64	Mutagenic	+	+	1.05
**12**	0.116	0.04	Yes	No	Yes	88.34	Mutagenic	+	+	0.64
**13**	0.144	0.03	Yes	No	Yes	79.76	Mutagenic	+	-	0.54
**14**	0.684	0.02	Yes	No	Yes	74.53	Mutagenic	-	+	-0.61
**15**	0.929	0.04	Yes	No	Yes	87.77	Mutagenic	+	+	0.78
**16**	0.946	0.04	Yes	No	Yes	84.39	Mutagenic	+	+	0.88
**17**	0.905	0.06	Yes	No	Yes	91.78	Non-mutagenic	+	-	0.97
**18**	0.984	0.03	No	No	Yes	100	Non-mutagenic	-	+	0.83
**19**	0.920	0.03	Yes	No	Yes	71.13	Non-mutagenic	+	-	0.67

*ABZ* Albendazole, *T*_*1/2*_ Half-life, *CYP2D6 & CYP3A4*:Inhibitor/substrate hepatotoxicity, *Carcino-test* Carcinogenicity positive or negative, *P-gp* P-glycoprotein substrate, *PPB* Plasma Protein Binding, *C*_*Brain/Blood*_ Penetration of the blood–brain barrier, *DLs* Drug Likeness Score

As it can be seen, the rules of Lipinski are not suitable for complicated natural products, concisely, only albendazole and compound (**14**) did not violate any of the Lipinski’s rule of five [70], however, two violations were observed for compounds (**1**, **7, 9**) therefore, may be good candidates for good availability, and consequently, corroborating its oral drug likeness properties, other compounds (**2–6, 8, 10–13, 15–19**) recorded 3 violations and were expected to be orally inactive (Table 4). Log *P*_*o/w*_ (octanol/water partition coefficient) an excellent parameter that measures the drug potency and plays a crucial role in the distribution of the drug in the body after absorption, all compounds were within an acceptable range of the log *P*_*o/w*_ values (–1.11 to + 4.50) suggesting good permeability through cell membrane. Also, a water-soluble compound facilitates the ease of formulation and oral administration to deliver a sufficient quantity of active ingredient [71]. All compounds showed predictions to be water soluble within the range of log *s* (–0.80 to –3.90), except compounds (**1, 7**, **12**) were moderately soluble with log *s* of (–5.08, –4.19 and –4.20) respectively. To clarify the absorption and passive transportation through biological barriers, TPSA parameter (Table 4) was predicted, which showed that only compound (**14**) displayed a good intestinal absorption with TPSA value 95.84 Å, however, other compounds exhibited relatively high TPSA values suggesting poor absorption through cell membrane.

Both of pharmacological activity and pharmacokinetics properties are essential targets for the drug discovery. One can observe from (Table 4) the absorption values HIA, P_CaCO2_ and P_MDCK_ that predicted for all compounds. Based on the fact that, HIA is a prime objective for the selection of a suitable oral drug-candidate [72], only compounds (**1**, **7**, **14**, **17**) having HIA values of > 50%, being close to that of **ABZ** (88%) and even better as predicted for compounds (**1**) and (**14**) with HIA values of 90.89 and 94.14% respectively, this reflects good oral drug absorption, whereas the other compounds (**2**–**6**, **8**–**13**, **15**, **16**, **18**, **19**) showed poor absorption.

Moreover, the cell models P_Caco2_ (nm/s) and P_MDCK_ (nm/s) have been used as trustworthy in vitro models for rapid screening permeability and to confirm oral drug absorption [73], as shown in (Table 4), only compound (**14**) revealed the best permeability closed to **ABZ** with 39.31 and 49.96 nm/s respectively, whilst, compounds (**1**, **6**, **7**, **9**, **12**, **13**) showed P_Caco2_ (nm/s) values > 20 nm/s. Furthermore, all compounds presented the lowest permeability P_MDCK_ values around 0.04 nm/s except compound (**14**) revealed 0.2 nm/s, as compared with 16.27 nm/s for **ABZ**. In addition, all compounds gave negative values of P_Skin_ accounted for its inappropriate utility for transdermal use with no risk present.

A drug to be distributed and partitioned in adipose and/or other tissues, it depends on its plasma protein binding (PPB) affinity. As appeared in (Table 5), compound (**18**) classified as the most potent with PPB 100% even more than **ABZ** 96.60%, while compounds (**1**, **6**, **17**) showed higher PPB strength with 94.87%, 91.22% and 91.78%, respectively. Compounds that are able to penetrate BBB can affect CNS, and produce neurotoxicity, which is a critical matter in the pharmaceutical field. All compounds (**1**–**19**) were observed to be having very low C_Brain_/C_Blood_ penetration values ranged between 0.02 – 0.65 (< 1), which accounted for their poor ability to pass across the BBB and/or collaterally affect the CNS, and hence classified as inactive [74]. P-glycoprotein (P-gp) is an efflux pump plays an important role in drug transport and distribution in many organs, so inhibition of P-gp as recorded by our compounds (**1–17**, **19**) can affect its protective function against xenobiotics, furthermore, it can reduce and/or increase the bioavailability of susceptible drugs [75].

About 90% of oxidative metabolic reactions depend on the CYP enzymes family (*i.e.,* 2C6, 3A4, …etc.), for a molecule to be involved in drug-drug interactions it should inhibits more CYP isoforms [76]. Our results demonstrated that all compounds (**1**–**19**) have negative prediction against CYP2D6 inhibition (Table 5), which is responsible for the metabolism of many drugs and toxic chemicals. Moreover, they were predicted to be inhibitors of the protein CYP3A4, which demonstrates their easy metabolization.

As appeared in (Table 5) toxicological properties which are important pharmaceutical requirements; including mutagenicity (Ames Test) and carcinogenicity (Mouse and Rat). Compounds (**2**, **4**, **6**, **7**, **11–16**) with positive prediction considered mutagenic, the other compounds (**1**,** 3**,** 5**,** 8**,** 9**, **17–19**) showed negative prediction and portend as non-mutagenic. Moreover, as appeared in (Table 5) compounds (**1**–**13, 15–17, 19**) showed prediction of positive carcinogenicity in mouse, and only two compounds (**14**, **18**) were predicted as negative carcinogenicity in mouse. Furthermore, the prediction of carcinogenicity in rat, revealed that the compounds (**1–3**, **6–12**, **14–16**, **18**) had positive prediction, whereas compounds (**4**, **5**, **13**, **17** and **19**) showed negative prediction.

The elimination half-life (*t*_½_) is the time it takes for the drug concentration to decrease by one-half, the larger the total clearance the shorter the half-life. It is a useful indicator of how fast a drug is removed from the body, as shown in (Table 5) all compounds (**1–19**) recorded long half-life *t*_½_. The selection of compounds as drug candidates were determined by a parameter called drug score. The higher the drug score value, the higher the compound chance of being considered a drug candidate, out of the list, compounds (**10**, **11)** recorded the best score value of 1.11 and 1.05, while compound **14** was the worst with score value of –0.61, the other compounds showed score value > 0.5.

## Conclusion

In the present study, it was proved that *B. indica* BuOH frac. can be used against the disease caused by *T. spiralis* infection, through lowering the parasite burden in the intestine and muscle tissues and alleviating muscular inflammatory reaction. Moreover, our results showed that *B. indica* BuOH frac. may be a potential anti-inflammatory agent in case of *T. spiralis* infection via depressing the expression of TNF-*α* in the muscle tissues. However, the experimental results clearly demonstrated that both *B. indica* BuOH frac., and albendazole were effective against intestinal adult worms and muscle larvae. In addition, TNF-*α* was controlled at low levels due to the bioactivity of *B. indica* BuOH frac., which indicated its powerful anti-inflammatory function in *T. spiralis* disease. Therefore, *B. indica* BuOH frac. may achieved anti-inflammatory effect by relieving the inflammatory response of the infected mice and eventually achieved the nematicidal effect of *T. spiralis*. Notably, although albendazole plays a more effective role in reducing the burden of *T. spiralis* larvae, due to its limitation in clinical use *B. indica* may be an ideal option to treat human trichinellosis.

The resulted promising anti-parasitic potential of *B. indica* BuOH frac. arouse our interest to go further to unveil its composition, with the help of LC/MS a total of **19** compounds (13 oleanolic-type triterpenoid saponins and 6 phenolics) were identified. A detailed in silico molecular docking investigation of the identified compounds (**1–19**) targeted certain protein receptors (PDB ID: 1OJ0, 2AZ5, *Ts*-CF1 and *Ts*-CRT). All ligands were observed to fit the binding pocket and interacted with the key amino acids at the active site, with different intermolecular interactions (HB-donor, HB-acceptor, H-π, π-π, π-cation) that energetically stabilizing ligands at the binding site of protein receptors, this noteworthy binding affinity reflects the inhibitory activity of the *B. indica* BuOH frac. against *T. spiralis* infection.

Our findings of the molecular docking are suggestive of *B. indica* BuOH frac. containing compounds (**1–19**) having the ability to affect the target enzyme systems in such a way as co-crystallized ligand and even better, which is an indicative for their potential anthelmintic activity against *T. spiralis* at different life stages. The ADMET properties as well as drug-likeness of all identified compounds were predicted, to stand on the suitable compounds that may be act as a future lead target.

## Supplementary Information

**Additional file 1.**

**Additional file 2.**

## Data Availability

All data generated or analyzed during this study are included in this published article and its supplementary information files.
